# Functional markers development and genetic diversity analysis in *Eucalyptus globulus* with emphasis in wood quality candidate genes

**DOI:** 10.1186/1753-6561-5-S7-P154

**Published:** 2011-09-13

**Authors:** Cintia Acuña, Pamela Villalba, Martín García, Esteban Hopp, Susana Marcucci

**Affiliations:** 1 Poltri Instituto de Biotecnología, CICVyA, INTA Castelar, CC 25, Castelar (B1712WAA), Argentina

## Background

*Eucalyptus globulus* is the most planted hardwood species for pulpwood in temperate regions. Genomic researches in *Eucalyptus* have increased the information available in DNA sequences public databases and several structural and regulatory genes involved in the cellulose and lignin pathways are known.

Functional genetic markers, while frequent in crop, are still scarce in forest species. Hence the detection and validation of SSRs in interesting genes to be used in future projects of marker-assisted breeding are needed.

The present study aimed the development of novel functional markers (SSRs) in ESTs and wood quality candidate genes (CG) from *Eucalyptus globulus*, and analyzes their potential for genetic diversity and individual identification studies.

We report the design of SSR primers flanking simple sequence repeats in ESTs and CG, the validation of a subset of randomly selected EST-SSRs using eight *E. globulus* genotypes and the screening of a sample of 60 trees with the polymorphic SSRs. Also, SSRs cross-transferability was tested in seven *Eucalyptus* species coming from three sections: *E. grandis*, *E. saligna* (section *Latoangulatae*); *E. globulus*, *E. dunnii*, *E. viminalis* (section *Maidenaria*); and *E. camaldulensis*, *E. tereticornis* (section *Exsertaria*).

## Material and methods

### Plant material

A total of 60 trees, each from a different OP family of *E. globulus*, were analyzed for their variability. These sixty trees represented major geographical races of the species’ natural distribution that were grown in a field trial in the Province of Buenos Aires, Argentina, between 1995 and 1997. For validation analyses one individual of each race (except Furneaux) was included.

A total of 47 individuals from seven species (including *E. globulus*) of the genus were sampled for the transferability analyses.

### Methods

Novel microsatellites were identified mainly by two different methods:

-SSR Mining software (GDR Server, http://www.rosaceae.org/bio/content?title=&url=/cgi-bin/gdr/gdr_ssr) on selected candidate gene sequences identified at the GenBank.

-SSRs detection from non redundant ESTs of *E. globulus* from GenBank: Annotations of these SSR-ESTs were based on the Gene Ontology (GO) (http://www.geneontology.org/) using Blast2GO (http://www.blast2go.org/) [1].

For validation and diversity analyses, amplification products were silver-stained or analyzed through an ABI3100 Genetic Analyzers (Applied Biosystems, USA) with fluorescent dyes respectively.

SSR statistics for determining number and frequency of alleles, effective number of alleles (N_e_), observed heterozygosity (H_o_) and unbiased expected heterozygosity estimates (UH_e_), fixation index (FI) and probability of identity (PI) were determined with the GenAlEx 6.4 program [2]. Tests for Hardy-Weinberg equilibrium were conducted using GENEPOP 4.0.10 [3]. Null allele frequencies were estimated with INEST software (Inbreeding/Null allele estimation) [4], using and Individual Inbreeding Model (IIM) with 10,000 iterations.

Structure analysis was explored through UPGMA and DAS (Shared allele distance) indices as well as Structure [5]software.

## Results

From 12,690 updated *E. globulus* EST database published in National Center for Biotechnology Information a total of 4,924 non-redundant sequences were identified. From these ones, 952 unigenes (19.3%) contained 1,140 SSRs. A new set of 979 primers was designed. The predicted functions of these EST-SSRs were adjudged, including biological process, molecular function and cellular component Gene Ontology (GO) categories.

Twenty four structural and regulatory candidate genes for wood quality carrying 29 SSR were indentified*.* Microsatellite sequences were located in UTR, introns and exons from candidate genes (CG) from: phenylpropanoid biosynthesis, cellulose biosynthetic process, hemicellulose metabolism, shikimate pathway, methionine metabolism, tubulin genes and the transcriptor factor LIM1.

Sixty five percent out of atotal of 85 SSR (56 EST-SSRs and 29 SSR containg GC) detected in this study were validated for actual PCR amplification of tree DNA samples in eight genotypes of *E. globulus*. From this assessment a total of 17 polymorphic EST-SSRs and 12 polymorphic CG-SSRs markers were obtained. These ones were selected for further analyses, so as to accurately estimate genetic information content in a larger sample of 60 non related trees represented major geographical races of the species’ natural distribution.

PIC, H_o_ and UH_e_ values varied over a wide range from around 0.02 to 0.9, whereas the allele number ranged from 2 to 16, with and average of 7.55.

A set of 49 *loci* (37 validated EST-SSRs (polymorphic and monomorphic) and 12 polymorphic CG-SSRs) were also tested for cross-transferability to other six *Eucalyptus* species (*E. grandis*, *E. saligna*, *E. dunnii*, *E. viminalis*, *E. camaldulensis*, *E. tereticornis*).A total of 33 out of the 49 validated markers in *E. globulus* amplified in the six other species and six markers amplified in at least other five.

Finally, the analyses of polymorphism and transferability of functional markers, enabled the selection of a set of 13 (7 EST-SSRs and 6 GC-SSRs) highly informative and transferable to six other species of *Eucalyptus*.

## Conclusions

The set of highly informative markers developed here will have potential use in studies of genetic diversity, taxonomy, gene mapping and will help the improvement of *Eucalyptus* trough the assisted selection.

Thirty percent of EST-SSRs (17 from 56) are expected to be polymorphic in *E. globulus* and 25% (14 polymorphic from 56) are expected also to be transferable to other six species (*E. grandis*,*E. saligna*, *E. dunnii*,*E. viminalis*,*E. camaldulensis* and*E. tereticornis*). Under these proportions, potentially more than 200 new EST-SSRs described here may contribute to the verification of synteny and collinearity between different *E. globulus* maps, and would allow the validation of gene and QTL positions in multiple pedigrees in the botanical sections *Maidenaria*, *Exsertaria*, and *Latoangulatae*, to which most of the commercially planted eucalypt species belong.
